# “Koehlers teardrop is not a reliable landmark for assessing the centre of rotation after Total hip arthroplasty” – a retrospective radiological study

**DOI:** 10.1007/s00402-023-04859-1

**Published:** 2023-04-26

**Authors:** Kristian Heinz, Dimitri Nowack, Rüdiger von Eisenhart-Rothe, Georgi Wassilew, Georg Matziolis, Steffen Brodt

**Affiliations:** 1 Orthopaedic Department, Jena Universitiy Hospital, Campus Eisenberg, Klosterlausnitzer Straße 81, 07607 Eisenberg, Germany; 2 Orthopaedic Department, Klinikum Rechts Der Isar, Munich Universitiy, Munich, Germany; 3 Orthopaedic Department, Greifswald Universitiy, Greifswald, Germany

**Keywords:** Total hip arthroplasty, Koehler’s tear drop figure, Centre of rotation

## Abstract

**Purpose:**

Various anatomical landmarks have become established in radiography for the assessment of cup positioning after total hip arthroplasty (THA). The most important one is Koehler's teardrop figure (KTF). However, there is a lack of data on the validity of this landmark, which is widely used clinically for assessing the centre of rotation of the hip.

**Method:**

A retrospective measurement of the lateral and cranial distance of the KTF to the centre of hip rotation was performed on the basis of 250 X-ray images of patients who had undergone THA. In addition, the dependence of these distances on pelvic tilt was determined in 16 patients by means of virtual X-ray projections based on pelvic CTs.

**Results:**

It was shown that the distance of the KTF from the centre of hip rotation in the horizontal plane is gender-dependent (men: 42.8 ± 6.0 mm vs. women: 37.4 ± 4.7 mm; *p* < 0.001) and age-dependent (Pearson correlation  – 0.114; *p* < 0.05). Furthermore, the vertical and horizontal distances are subject to variation depending on height (Pearson correlation 0.14; *p* < 0.05 and 0.40; *p* < 0.001, respectively) and weight (Pearson correlation 0.158; *p* < 0.05). The distance between the KTF and the centre of hip rotation varies slightly depending on pelvic tilt.

**Conclusion:**

The KTF is not a sufficiently valid landmark for assessing the centre of rotation after THA. It is influenced by many different disturbance variables. However, it is largely robust against changes in pelvic tilt, so that it can be used as a reference point when comparing different intraindividual radiographs to assess the change in the centre of rotation due to implantation or to detect cup migration.

## Introduction

**Fig. 1:**
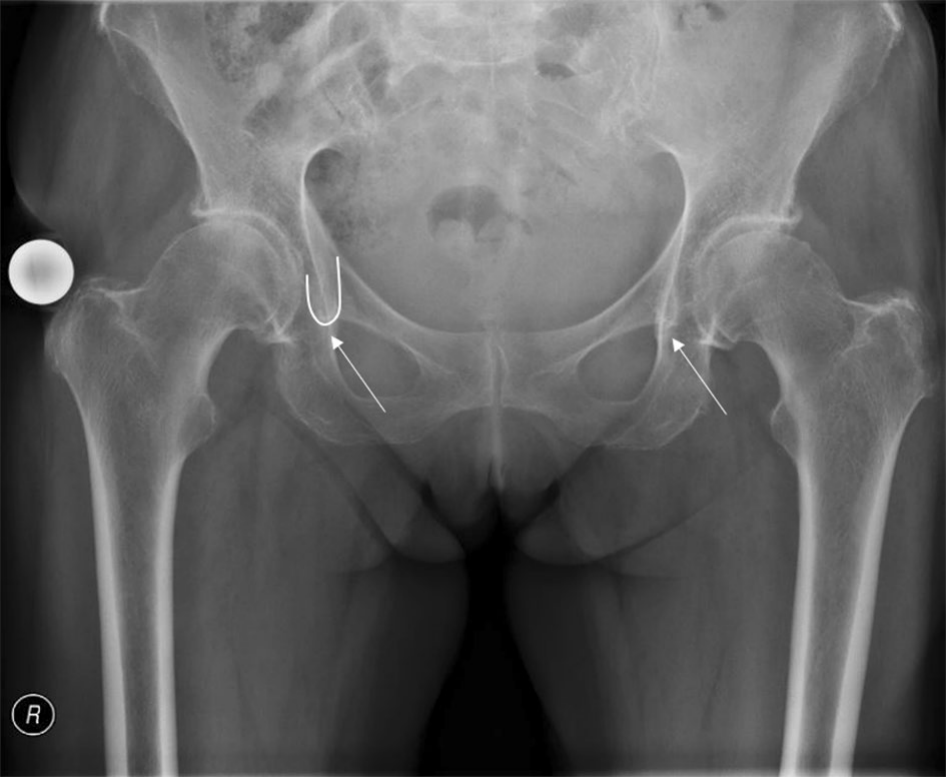
Pelvic survey a.p. showing KTF (arrows right and left), and highlighted for illustration (on the patient’s right *side)*

Total hip arthroplasty (THA) is one of the most efficient surgical procedures worldwide [1]. Total hip replacement not only reduces pain, but it also fully restores the function of the affected limb. The implant survival rate is about 93% after 20 years [1–3]. Non-physiological positioning of the centre of rotation (medialisation, lateralisation, cranialisation, caudalisation) can lead to a muscular deficit, such as a positive Trendelenburg sign, due to insufficient contraction of the gluteus medius and gluteus minimus muscles [4–6]. Furthermore, it has been shown that a femoral head that is not implanted in an anatomically correct position can lead to early loosening of the prosthesis [7] and increased migration, with a subsequent need for prosthesis revision [8–11]. If the cup is placed too medially, there is a risk of offset loss. If the centre of rotation (COR) chosen is too high, this potentially leads to leg shortening. Although both offset and leg length can be compensated for by appropriate selection and positioning of the stem, this results in non-physiological hip contact forces and insufficiencies of the pelvitrochanteric musculature [4–6]. The goal of cup positioning must therefore be to achieve the most anatomical reconstruction possible [12–14]. In the evaluation of the postoperative COR, anatomical "landmarks" such as the bony acetabular roof and the tear drop have proven useful. The Koehler's teardrop figure (KTF) was first described by Köhler in 1931 [15]. It is the most commonly used anatomical structure for positioning the artificial cup in THA. It has no direct anatomical correlate, but is created by the radiographic superimposition of different anatomical structures in the anterior–posterior ray path and forms a U-shaped figure along the medial surface of the acetabulum (Fig. 1).

In various publications, the most caudal points of the two teardrop figures are connected vertically from the KTF to determine the centre of the femoral head [7, 8]. On the resulting line, a perpendicular line is drawn in the direction of the centre of the femoral head and measured. In the horizontal direction, the anatomical reference structure is also the furthest caudal point, depending on the publication [16–20]. If the centre of the femoral head is known by measurement, subsequently prepared radiographs can also be used to draw conclusions regarding protrusio acetabuli or acetabular migration after incorporation of a THA [42]. Massin et al. [21] recommended the most caudal edge of the teardrop figure as a reference point for determining the extent of acetabular migration in both vertical and horizontal directions. The KTF is represented two-dimensionally on the radiograph. Its use as an anatomical landmark presumes a constant relation on a two-dimensional pelvic anterior–posterior X-ray. Despite its wide use, this consistency has not been proven and discussed in the literature yet. To date, it remains unclear, if the KTF is a valid landmark for evaluating the position of the centre of rotation after THA. The objective of the present paper was to investigate, if the KTF can be used as an anatomical reference for assessing the correct center of rotation (COR) for cup placement in THA in regular anatomies (primary osteoarthrosis). Are there any patient-specific variables (e.g. age or height) that influence the presentation of the KTF? Does the pelvic tilt lead to a changed representation of the KFT and thus to an incorrect assessment of the COR reconstruction? The questions should be clarified in the present work.

## Materials and methods

The present study included 250 patients with 250 standardised pelvic survey radiographs taken from 09 to 12/2020 prior to planned THA. Only radiographs of patients with completed skeletal maturation and anatomically correct configuration of the pelvis were used for the study. The hip joint had to fulfil the following characteristics:

- the femoral head had to be spherical (femoral head ratio < 1.2), with the spherical centre representing the COR,
- patients with primary osteoarthritis of the hip were selected,
- no dysplastic changes of the hip joint were allowed.

**Fig. 2:**
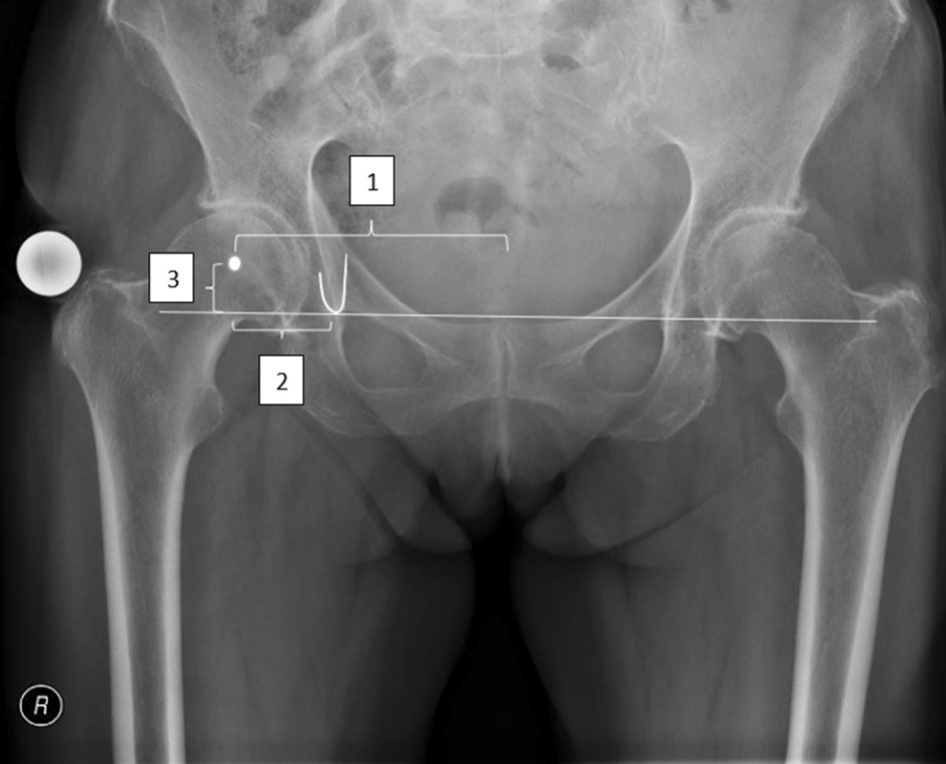
Pelvic survey a.p. with anatomical landmarks (∆: distance symphysis—COR, X: distance KTF—COR, + : distance COR—horizontal line through the most caudal points of the KTFs*)*

For our data analysis, we used a low pelvic overview radiographs in the anterior–posterior ray path for all patients with a reference sphere (25 mm) according to in-house standards (see Fig. 2). This ensured a consistently valid measurement process. In Fig. 2, the right hip joint should serve as an example for the parameters collected for data analysis. First, the center of rotation of the examined hip joint was located (circle in the right femoral head). Then, the caudal tip of both KTFs was fixed and connected as a horizontal line. Now, a perpendicular was placed from this horizontal line through the COR. From this point of intersection, the mediolateral (X) and craniocaudal ( +) distance (KTF – COR) was measured. The measurements were made using the PACS viewer AGFA Impax FX and AGFA XERO Viewer 8.1.2.

In addition, virtual radiographs of 16 pelvic CTs (16 patients) were taken. The pelvic tilt was changed between  – 30° and + 30° in 10° steps and the distance of the COR of the hip to the KTF was determined using the same methodology.

The data obtained were first entered in an Excel table (Microsoft) and then statistically analysed using SPSS (SPSS GmbH Software Munich, Germany). Means and standard deviations of the measured values were determined. As a visual measure of validity and clinical usability, ellipses were drawn around the COR with the single and double standard deviation as diameters. This corresponds to a probability of 68% and 95%, respectively, that the actual centre of rotation lies within the ellipse, given a normal distribution of the data.

A normal distribution was found for the mediolateral and craniocaudal distance of the KTF to the COR in both the Shapiro–Wilk test and the Kolmogorov–Smirnov test. Thus, the t-test for normally distributed data was used.

Dependencies of these data on patient-specific factors such as gender, height and age were tested using *t*-test and Pearson correlation at a significance level of 0.05. Cronbachs Alpha was calculated to determine inter- and inter-rater reliability. Three investigators (K.H., S.B., G.M.) performed all measurements independently. One investigator (K.H.) performed all measurements additional two times.

## Results

Among the subjects, 132 were male (52.8%) and 118 female (47.2%). The age at the time of radiography was between 39 and 84 years (67.1 ± 9.7 years) in the female patients. In the male patients, the average age at this time was 63.4 ± 11.0 years, with a range of 35–87 years.

**Fig. 3:**
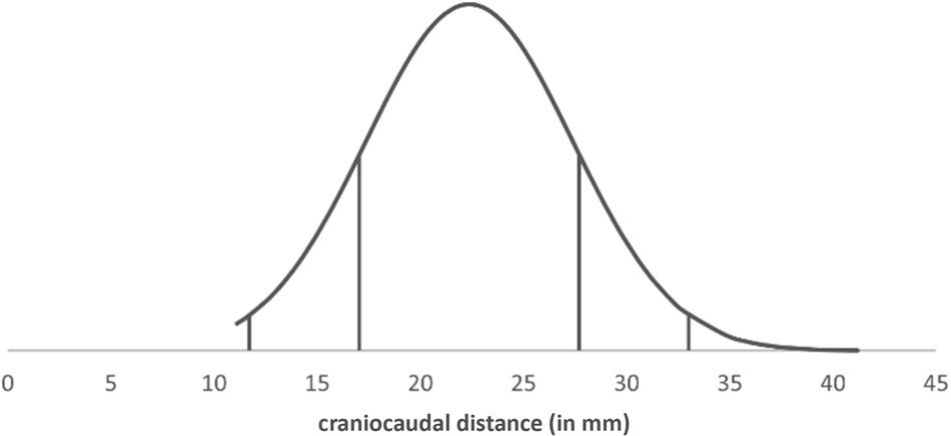
Normal distribution of craniocaudal distance KTF/COR (in mm)

**Fig. 4:**
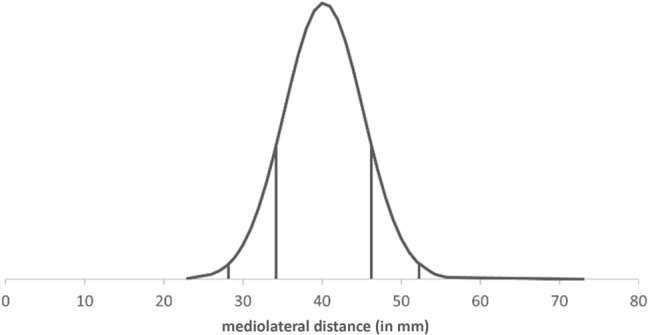
Normal distribution of mediolateral distance KTF/COR (in mm)

The measured values for the vertical and horizontal distance of the KTF to the COR showed a normal distribution in each case (Figs. 3 and 4). Figures 3 and 4 show interpolated lines of our patient cohort.

**Fig. 5:**
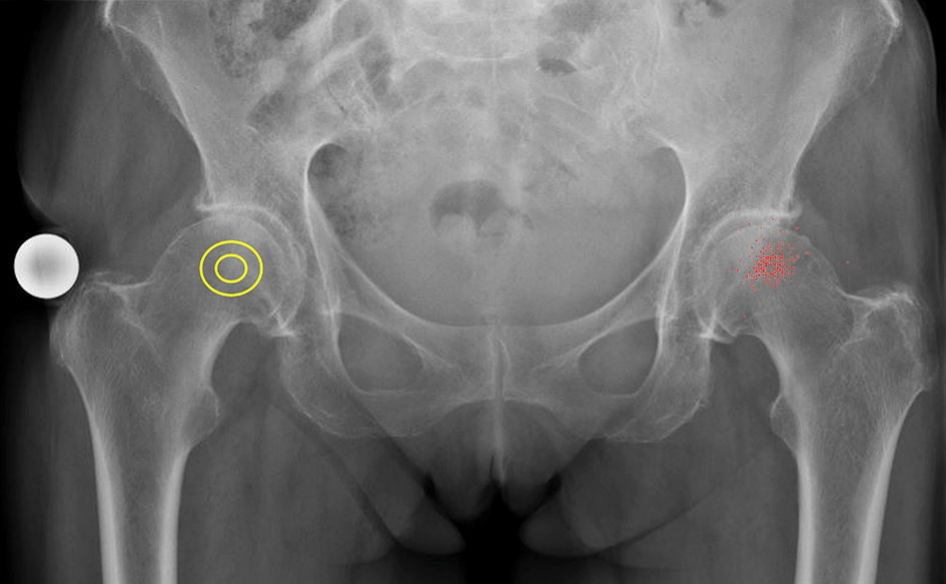
Pelvic survey a.p. showing the centres of rotation of the patient collective

In the patient collective, the average vertical distance was determined to be 22.4 ± 5.3 mm and the horizontal distance 40.2 ± 6.0 mm (Fig. 5). In Fig. 5 the pelvic radiogram should represent the COR distribution of our patient cohort. For this purpose, the magnification and resolution of the X-ray was exactly scaled. Therefore, it was possible to depict the corresponding data cloud in the left hip. On the right side, the small sphere represents the 68% and the big sphere the 95% confidence interval of our patient cohort.

Furthermore, gender-specific differences were found in the horizontal distance of the COR to the KTF (men: 42.8 ± 6.0 mm vs. women: 37.4 ± 4.7 mm; *p* < 0.001), height (men: 1.8 ± 0.1 m vs. women: 1.7 ± 0.1 m; *p* < 0.001), weight (men: 93.8 ± 14.9 kg vs. women: 80.4 ± 18.0 kg; *p* < 0.001) and postoperative cup diameter (men: 56.7 ± 2.7 mm vs. women: 51.1 ± 4.9 mm; *p* < 0.001). Age was shown to correlate negatively with the distance centre of rotation-symphysis (*p* < 0.001) and centre of rotation-KTF (*p* < 0.005). There were also positive correlations between height and the distance between the COR and the symphysis (*p* < 0.001), the vertical distance between the COR and the KTF (*p* < 0.05) and the horizontal distance between the COR and the KTF (*p* < 0.001).

Intra- and inter-rater reliability was excellent (> 0,9) for all measurements: Intra-rater reliability was 0,961 for the distance between symphysis and COR, 0,986 for the distance between horizontal axis and COR, and 0,983 for the distance between KTF and COR. Inter-rater reliability was 0,946 for the distance between symphysis and COR, 0,971 for the distance between horizontal axis and COR, and 0,973 for the distance between KTF and COR.

In addition, 16 pelvic CT examinations were evaluated in which a pelvic tilt between  – 30° and 30° was set and the distance COR-KTF was measured.

**Fig. 6:**
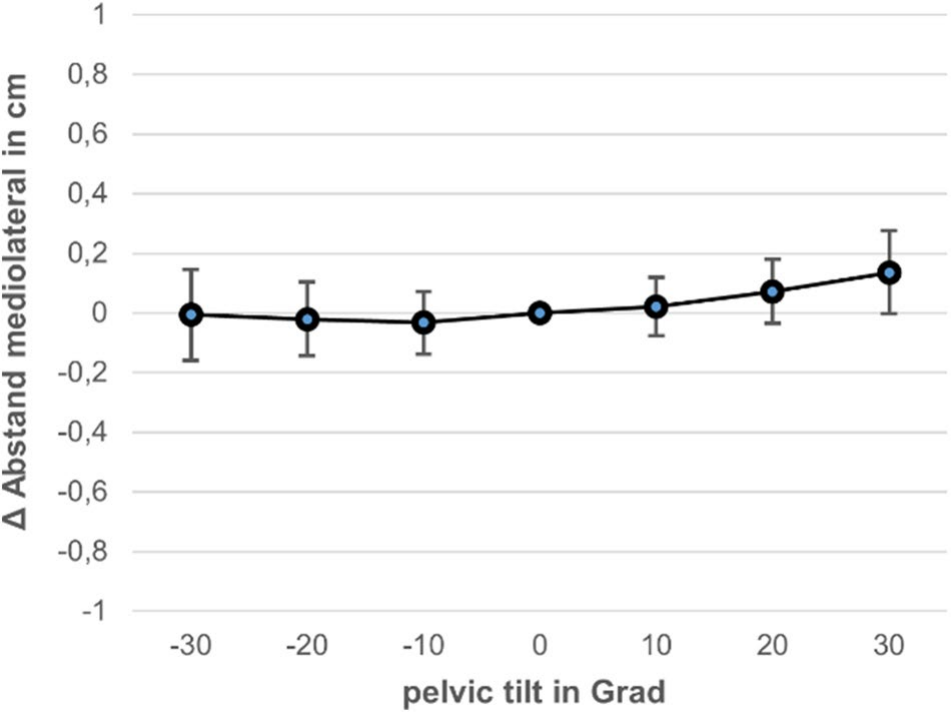
Change in the mediolateral distance KTF/COR (in mm), dependent on pelvic tilt (in degrees)

Nine male (68.6 ± 9.3 years) and seven female (68.6 ± 5.7 years) subjects were examined. It was shown that the distance between the caudal tip of the KTF and the COR of the hip showed only slight changes of less than 2 mm as a result of pelvic tilt (Fig. 6). These were also only significant at the most extreme angles considered of 20° (0.1 ± 0.1 cm; *p* < 0.001) and 30° (0.2 ± 0.1 cm; *p* < 0.001). In the range between ± 10° pelvic tilt, which is relevant in routine radiology, the deviation was less than 1 mm, so that the distance between the KTF and the centre of the hip can be determined reproducibly in the pelvic survey image.

## Discussion

The aim of this work was to examine whether the KTF represents a valid anatomical landmark to assess the reconstruction of the COR after THA in primary osteoarthritis. Furthermore, we wanted to prove possible variables that influence the representation of this landmark.

The main result of the present study is that the KTF is not a sufficiently valid landmark for assessing the COR after THA. It could be shown that this anatomical landmark is influenced by age, height, weight and gender as well as—to a markedly lesser extent—the pelvic tilt.

Implantation of a total hip replacement is a successful and frequently performed hip surgery procedure [24, 26], whose second most common complication after aseptic loosening is prosthesis dislocation [27]. Incorrect cup position has a decisive influence on the development of impingement syndrome, wear of the articular pairing and the occurrence of osteolytic processes with subsequent implant loosening and, thus, on the outcome of the procedure [28]. Consequently, the anatomically correct position of the COR is crucial for optimising cup positioning and thereby postoperative functionality after THA. The preoperative pelvic survey radiograph serves as a support for the surgeon during implantation. By using the KTF to calculate the COR, an anatomical structure is selected that lies in the same plane as the prosthesis [29]. However, especially in the case of pronounced defects of the acetabular bed, it is often no longer possible to identify this anatomical structure. In these cases, alternative means of determination must be used. One of the main problems, however, is that there is a lack of standard values for determining the anatomical COR. The values determined in the paper of John et al. [30] take the KTF as a reference point, the very structure that is often no longer recognisable in the case of pronounced defects of the acetabular bed. For this frequently occurring situation, there are currently no methods available for determining the COR preoperatively. This makes exact planning of such an operation difficult.

Apart from this, according to Bowerman et al. [31], even slight rotations of the pelvis influence the distance from the teardrop figure to the COR, which in turn leads to increasing measurement inaccuracy. Many studies investigated pelvic tilt and its consequence for the diagnostic and therapeutic evaluation of cup orientation parameters [23, 33–36]. Acetabular orientation is influenced by pelvic tilt and is consequently part of a dynamic interaction [22, 25, 37, 40]. With a maximum difference of less than ten degrees between supine and standing body positions, pelvic tilt shows relative, individual consistency [37–39]. Another frequently used structure, Köhler’s line, is located posterior to the acetabulum [32]. The working groups led by Gates III [16] and Goodman [9] were able to demonstrate that the KTF has a higher consistency than Köhler’s line during rotation of the pelvis. According to Rusotti et al. [20], an increase in pelvic flexion from 0° to 10° in pelvic survey images is already associated with an increase in the height localisation of the centre of two millimetres, measured from the KTF.

However, if the pelvis is markedly rotated around one axis in the pelvic survey image, it should be taken into consideration that the KTF is the reference structure to the bony acetabulum that shows the highest consistency on rotation in both the sagittal and coronal ray paths [41].

The present data show that the KTF is not a sufficiently valid landmark for assessing the centre of rotation after THA. At best, it is suitable as a reference for intraindividual comparison of radiographs.

## Data Availability

I do not wish to share my data because they involve the privacy of patients in our hospital. But the datasets used and/or analyzed during the current study are available from the corresponding author on reasonable request.
